# Reliability of biomarkers of sepsis during extracorporeal therapies: the clinician needs to know what is eliminated and what is not

**DOI:** 10.1186/s13054-020-03277-8

**Published:** 2020-09-11

**Authors:** Patrick M. Honore, Sebastien Redant, David De Bels

**Affiliations:** ICU Department, Centre Hospitalier Universitaire Brugmann, Place Van Gehuchtenplein,4, 1020 Brussels, Belgium

**Keywords:** Removal of biomarkers, CRRT, Procalcitonin, Presepsin, MR-pro-ADM, Endocan, Pentraxin-3, Heparin binding protein, Osteopontin

## Background

The evolution of renal replacement therapy (RRT) techniques, and the increasing number of critically ill patients receiving extracorporeal therapies, has presented clinicians with a significant problem: if biomarkers are removed by RRT, can they still be considered reliable in their role of guiding diagnosis and treatment?

**Fig. 1:**
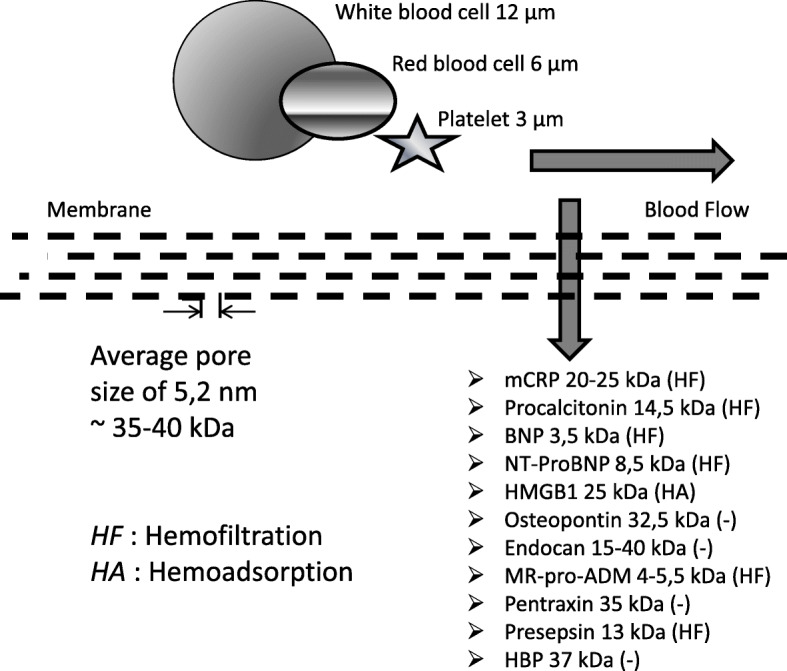
Biomarker molecular weight and removal by CRRT membranes

The most commonly used RRT techniques in intensive care units (ICUs) can be classified into three categories: continuous renal replacement therapy (CRRT), intermittent hemodialysis (IHD), and hybrid techniques such as those performed with sorbent devices and plasma exchange (PE). These techniques remove substances from the plasma via convection, adsorption, or a combination of the two. Various factors determine the degree of removal, including molecular weight (MW) and charge, and the type of membrane and RRT technique used. IHD has a cut-off of 5 kDa in most cases and the risk of eliminating biomarkers is small. For CRRT, the cut-off value of the membranes is about 35 kDa, and as a result, filtration of a significant number of biomarkers may occur. New highly adsorptive membranes (HAMs), such as the acrylonitrile 69-surface treated (AN69-ST), are being used more frequently in ICUs [1]. This means that biomarkers with a MW above 35 kDa, while not removed by convection, may potentially be removed in a significant quantity by adsorption. With hybrid devices like CytoSorb, removal of hydrophobic substances with a MW up to 55 kDa occurs via selective binding [2]. PE has a cut-off of 1000 kDa and removes not only biomarkers but also a range of other substances including clotting factors and immunoglobulins. Clearance of a substance cannot always be predicted from MW and RRT membrane characteristics alone, highlighting the need for further studies to determine biomarker levels pre- and post-device for different CRRT techniques. For example, the relatively small MW (25 kDa) of high mobility group protein B1 (HMGB1), a damage-associated molecular pattern (DAMP) and marker of outcome, in theory does not prohibit its removal by convection. However, HMGB-1 is not eliminated by convection and is only effectively cleared through adsorption by HAMs like AN69-ST [3]. This occurs because it has a flat shape, and this prevents its passage through a CRRT membrane, despite its small MW. The degree of biomarkers removal by RRT, with the consequent effect on their serum levels, is essential information for clinicians (Fig. 1).

## Biomarkers eliminated by CRRT and sorbents

*C-reactive protein* (CRP) is the most commonly used biomarker of inflammation. While often thought of as a pentamer with a MW of 125 kDa, CRP is predominantly present as a monomer (mCRP, MW 22–25 kDa) in the blood of septic patients and as such is removed by all forms of CRRT. Substantial amounts can also be eliminated via adsorption, by both conventional CRRT membranes and the CytoSorb device [4].

*Procalcitonin* (PCT), a biomarker used to detect (and exclude) the presence of infection and to monitor response to treatment, has a MW of 13.5 kDa and has been detected in the ultrafiltrate of patients undergoing CRRT [5]. Most of the PCT is eliminated by convection, but adsorption also contributes to elimination during the first hours of treatment [5].

*B-type natriuretic peptide* (BNP) and *N-terminal pro-BNP* (NT-proBNP), biomarkers of cardiac dysfunction and outcome in sepsis, are also highly likely to be easily cleared by CRRT given their low MWs (3.5 kDa for BNP and 8.5 kDa for NT-proBNP) [6].

*Mid-regional pro-adrenomedullin* (MR-pro-ADM), a biomarker of sepsis severity and response to treatment, has a MW between 4 and 5.5 kDa, and its plasma concentration has been shown to decrease by 45–65% if a high-flux membrane is used [7].

Recently, *presepsin* has also been identified as a diagnostic biomarker of sepsis [8]. It has a MW of 13 kDa, which theoretically means that it could be subject to significant convective elimination.

Given that RRT artificially decreases creatinine levels, a patient under RRT should be considered as having the full acute kidney injury in any score.

## Biomarkers not eliminated by CRRT or sorbents but needing further investigation

*Endocan* is a diagnostic and prognostic biomarker for sepsis and acute respiratory distress syndrome [9]. CRRT with a membrane cut-off of 35 kDa is unlikely to remove endocan (MW 40 kDa), but removal may possibly occur by adsorption when HAMs are used [10].

*Pentraxin 3* (PTX3), a marker of sepsis severity and a diagnostic marker for ventilator-associated pneumonia [11], has a MW of 35 kDa and thus, in theory, can be removed by CRRT. However, a recent study demonstrated little or no clearance or absorption by the filter during CVVH [12].

*Heparin binding protein* (HBP), a predictor of sepsis-induced organ dysfunction [13], has a MW of 37 kDa and as such should not be removed by convection. HBP has been detected in the effluent of patients undergoing CRRT, without a consistent decrease in plasma levels [14]. Studies are needed to investigate whether adsorption is possible.

*Osteopontin* (OPN) is a predictor of outcome in critically ill patients [15]. A highly negatively charged protein with a MW of 32 kDa, osteopontin can theoretically be removed by CRRT, but at this time evidence is lacking.

Table 1 summarizes all biomarkers described in this review with their MW, ability to be removed by convection and/or adsorption, and whether a study focusing on removal via RRT has been performed.

**Table 1:** Biomarker molecular weight, potential removal by CRRT or sorbents, and summary of the available studies and the studies that need to be realized of levels in the context of RRT

Biomarker	Molecular weight (kDa)	Elimination by CRRT	Elimination by sorbents	Existing studies	Studies needed
mCRP	20–25	+	+	+	+
PCT	14.5	+	+	+	+
BNP	3.5	+	+	+	+
NT-ProBNP	8.5	+	+	+	+
HMGB-1	25	+ (adsorption only)	+	+	–
OPN		–	+	–	+
Endocan	40	–	+	–	+
MR-pro-ADM	4–5.5	+	–	–	+
PTX3	35	–	+	+	+
Presepsin	13	+	+	+	+
HBP	37	–	+	–	+

*kDa* kilodalton, *CRRT* continuous renal replacement therapy, *mCRP* monomeric C-reactive protein, *PCT* procalcitonin, *NT-ProBNP* N-terminal pro-hormone of brain natriuretic peptide, *OPN* osteopontin, *HMGB-1* high mobility group protein B1, *MR-pro-ADM* mid-regional pro-adrenomedullin, *HBP* heparin binding protein

## Conclusions

It is likely that many sepsis biomarkers may be removed by convection, and therefore, their reliability as markers in patients undergoing CRRT is under question. Furthermore, the increasing use of HAMs makes the removal of many biomarkers even more likely. It is possible that some biomarkers may still have utility in the role of guiding diagnosis and treatment of critically ill patients on CRRT; however, further studies exploring biomarker elimination by CRRT are needed to confirm this. The development of new reference ranges for biomarkers in the setting of RRT would also be an interesting avenue of study. Beyond their utility as biomarkers, there are still many other questions to answer, such as whether removal of these, and other, substances by CRRT may result in benefit or harm.

## Data Availability

Not applicable.

## Abbreviations

RRT: Renal replacement therapy
ICU: Intensive care unit
CRRT: Continuous renal replacement therapy
IHD: Intermittent hemodialysis
PE: Plasma exchange
MW: Molecular weight
HAM: Highly adsorptive membrane
AN69-ST: Acrylonitrile 69-surface treated
kDa: Kilodalton
HMGB1: High mobility group protein B1
DAMP: Damage-associated molecular pattern
CRP: C-reactive protein
mCRP: Monomeric form of CRP
PCT: Procalcitonin
BNP: B-type natriuretic peptide
NT-proBNP: N-terminal prohormone of brain natriuretic peptide
MR-pro-ADM: Mid-regional pro-adrenomedullin
PTX3: Pentraxin-3
HBP: Heparin binding protein
OPN: Osteopontin
